# Unraveling the Heat- and UV-Induced Degradation of Mixed Halide Perovskite Thin Films via Surface Analysis Techniques

**DOI:** 10.1021/acs.langmuir.3c03816

**Published:** 2024-05-23

**Authors:** Pei-Chen Huang, Ting-Jia Yang, Chia-Jou Lin, Man-Ying Wang, Wei-Chun Lin

**Affiliations:** Department of Photonics, National Sun Yat-sen University, Kaohsiung City 80424, Taiwan (R.O.C.)

## Abstract

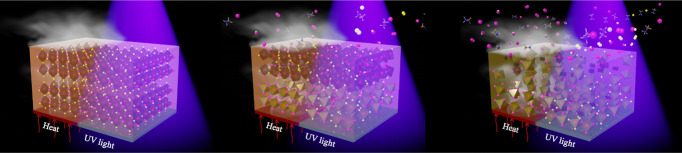

In recent years, organic–inorganic hybrid perovskite materials have become one of the most promising materials in the new generation of solar cells. These perovskites can provide excellent photoelectric properties after a simple fabrication process. Although perovskite solar cells have achieved high power conversion efficiency, instability concerns regarding material exposure to heat, moisture, air, and UV light present hindrances to commercialization. In this study, three kinds of perovskites (MAPbI_3_, MAPbI_3–*x*_Br_*x*_, and MAPbI_3–*x*_Cl_*x*_) were used to investigate the crystal stability upon exposure to heat and UV light. SEM, XRD, and FTIR were used to observe the surface morphology, crystal structure, and functional groups of the perovskite thin films. XPS was used to examine the surface composition and chemical state of the perovskite thin films under different conditions. Among these three types of perovskites, it was found that the MAPbI_3–*x*_Br_*x*_ crystal demonstrated the best stability. ToF-SIMS was used to confirm the molecular distribution of the MAPbI_3–*x*_Br_*x*_ films upon exposure to heat and UV light at different depths. ToF-SIMS revealed that [Pb]^+^ and [PbI]^+^ aggregated at the interface between the perovskite and ITO substrate after 14 days of thermal treatment. On the other hand, [Pb]^+^ and [PbI]^+^ were distributed uniformly after 3 days of UV exposure. This study systematically analyzed and revealed the thermal- and UV-induced degradation process of three perovskite films by using surface analysis techniques. It was concluded that bromine-doped perovskite films had better stability, and UV light caused more severe damage than heat.

## Introduction

Organic–inorganic hybrid halide perovskites have attracted widespread attention in recent years due to their outstanding characteristics, such as large absorption coefficients,^1,2^ tunable band gaps,^3^ high charge carrier mobilities,^4^ and long charge diffusion lengths.^5,6^ Unlike other kinds of photovoltaics, perovskite solar cells (PSCs) have the advantages of simple manufacturing processes,^7^ low cost,^8,9^ and high power conversion efficiencies.^8,10^ Therefore, PSCs are viewed as one of the most promising types of photovoltaic devices and are undergoing rapid development. In 2023, the highest power conversion efficiencies of PSCs reached 26.1%.^11^ Despite the excellent prospects of PSCs, there are still several challenges, such as stability,^12−15^ defects,^16,17^ and the method for large-scale manufacture,^18^ which need to be overcome before commercialization. Among these challenges, the long-term stability of PSCs is a critical issue that must be addressed. It is well-known that perovskites are sensitive to different environmental conditions, such as heat,^19−21^ moisture,^22^ air,^23^ and light exposure.^24−26^ These environmental factors facilitate the decomposition of perovskite crystals. Among these environmental factors, heat and illumination are two of the most influential factors and must be seriously considered since they inevitably occur during the operation of devices. Accordingly, many studies on the degradation mechanism of perovskites have been published in the effort to determine the degradation pathways of perovskite crystals and provide ideas to improve the design of PSCs.^13−15,21−25^ For example, Kim et al. confirmed that the surface structure of MAPbI_3_ perovskite films changes to an intermediate phase and decomposes to CH_3_I, NH_3_, and PbI_2_ after both a short exposure (20 min) to heat stress at 100 °C and a long exposure (>1 h) at 80 °C.^21^ In their study, they observed changes in the orientation of CH_3_NH_3_^+^ organic cations concerning the substrate in the intermediate phase, which might be linked directly to the thermal degradation processes in MAPbI_3_ perovskites.^21^ Juarez-Perez et al. presented reversible and irreversible photodecomposition reactions of methylammonium lead iodide perovskites (MAPbI_3_).^25^ Decomposition reactions of MAPbI_3_ under illumination and mild heating (40–80 °C) are listed below:
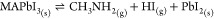
1
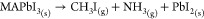
2
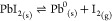
3

Equations 1 and 3 describe reversible pathways, while eq 2 does not.^25^ This has suggested to scientists that limiting reaction 2 could facilitate the self-healing of PSCs, which might prolong the lifetime of devices.^25^ Several literature works have investigated the possibility of the reversible and irreversible reactions by providing a suitable atmosphere (CH_3_NH_2_ and HI), which leads to reform perovskite crystals.^27−31^ Some practical strategies to increase the lifetime of PSCs have been proposed based on the understanding of the degradation phenomena of perovskites. It is shown that the decomposition of perovskites caused by moisture and oxygen could be simply prevented by encapsulation.^32−35^ This method could also suppress the leakage of volatile products released from the perovskite, making the recovery process possible.^33,34^ However, analogous strategies could hardly offer a solution to heat-induced and light-induced degradation since only a few studies have tried to address these issues. Therefore, a deeper understanding of heat- and light-induced degradation is necessary.

Organic–inorganic hybrid halide perovskites are materials with a crystal structure following the formula ABX_3_, which consists of an A-site cation, a B-site cation, and a halide anion (X-site). The stability of perovskite crystals could be improved with better photovoltaic properties by inducing different A-site cations^36,37^ or halide elements.^38−40^ Several studies have found that tuning the composition of the perovskite apparently changes the PSC’s characteristics.^41−49^ With a small amount of bromide replacing iodine in the composition of MAPbI_3_, a mixed halide perovskite (MAPbI_3–*x*_Br_*x*_) was shown to have cell efficiencies boosted to 20.7% with a controllable band gap from 1.5 to 2.3 eV.^42^ Sutter-Fella et al. also showed that the internal luminescence quantum yield of the wide band-gap perovskite MAPbI_3–*x*_Br_*x*_ reaches impressive values up to 30%.^43^ For a mixed halide perovskite (MAPbI_3–*x*_Cl_*x*_), DFT calculations demonstrated interfacial chloride-induced band bending, which may improve the charge collection efficiency of the device and possibly affect the recombination pathways of hot carriers.^46^ Cao et al. indicated that the performance of MAPbI_3–*x*_Cl_*x*_ solar cells was significantly improved compared to that of MAPbI_3_ solar cells without chloride incorporation.^49^ Similar results were found when examining the degradation phenomena of MAPbI_3–*x*_Br_*x*_, MAPbI_3–*x*_Cl_*x*_, etc.^44,50−53^ Ruan et al. revealed that MAPbI_3–*x*_Br_*x*_ underwent light-induced decomposition due to phase segregation from the initial I-rich domain under illumination.^50^ By adding bromide and adjusting the composition of the halide, the stabilization of the I-rich phase of MAPbI_3_ could be improved.^50^ Liao et al. reported a hot-casting process with controlled Cl^–^ incorporation that improves the device stability by passivating the reaction between I^–^ and the silver electrode.^52^ All these results indicate that perovskites show distinct differences in behavior when Br or Cl is mixed into the crystals. However, few studies have been focused on investigating the stability of different mixed halides from the perspective of surface chemistry.

The surface analysis technique is a powerful method that provides diverse information regarding precise elemental composition,^54,55^ chemical states,^54,55^ and molecular distribution.^22,56^ The most intense interaction between perovskites and external environmental factors is from the outermost surface (∼10 nm) of the thin films. Such information from the outermost surface would likely to be obtained only by surface analysis methods. X-ray photoelectron spectroscopy (XPS) is one of the mainstream surface analysis instruments, which provides information about chemical states and composition from ∼6–7 nm depths of the thin film.^54,55^ XPS could help to track chemical changes in the perovskite crystals during degradation. A time-of-flight secondary ion mass spectrometry (ToF-SIMS) is another surface analysis instrument that can be used to investigate the molecular distribution of the specimen.^22,56^ In combination with cluster ion sputtering, it is possible to construct slide-and-view 3D molecular distribution depth profiles at the nanoscale.^22,56^ Therefore, it is exciting to perform a cross comparison of the degradation phenomena of different kinds of perovskite crystals by using the surface analysis instruments mentioned above. In fact, surface analysis methods have played an important role in the study of perovskites^57−60^ and have provided unique information for scientists. In this study, the degradation phenomena of three kinds of perovskite films (MAPbI_3_, MAPbI_2.91_Br_0.09_, and MAPbI_2.25_Cl_0.75_) upon exposure to heat (according to the IEC 61646 standard) and UV light (254 nm) were investigated via conventional analysis techniques, such as X-ray diffraction (XRD), Fourier transform infrared spectroscopy (FTIR), and scanning electron microscope (SEM). XPS was primarily used to analyze the compositional change and monitor the chemical states during degradation. ToF-SIMS was used to confirm the observations from XPS with a depth-dependent distribution of species on the most stable mixed halide perovskite (MAPbI_3–*x*_Br_*x*_) upon exposure to heat and UV light over time. The XPS spectra demonstrated that the formation of metallic Pb^0^, PbO, and great loss of nitrogen are the primary ways to cause decomposition of the perovskite crystals. The ToF-SIMS analysis of the MAPbI_3–*x*_Br_*x*_ films proved the formation of molecules speculated from XPS data. The ToF-SIMS depth profiles further found that [PbBr]^+^ aggregated at the interface region between perovskite and ITO.

## Experimental Section

### Perovskite Fabrication

A series of preparation steps were applied to ITO substrates before thin film fabrication. To remove surface contamination from the substrate, cleaning detergent, DI water, ethanol, acetone, and isopropanol were used to conduct an organic solvent cleaning process in an ultrasonic tank for 15 min. The substrates were then dried and stored before being used. The PbI_2_ precursor solution was prepared by dissolving 460 mg/mL lead iodide powder in *N*,*N*-dimethylformamide (DMF) and then stirring overnight at 70 °C. A methylamine iodide (MAI) precursor solution was prepared by dissolving 40 mg/mL MAI powder in isopropanol (IPA) and then stirring at room temperature (∼25 °C). 50 mg of MAI powder and 10.4 mg of methylamine bromine (MABr) powder were dissolved in 1 mL of IPA at room temperature to produce an MAI/MABr mixture precursor solution. Lead chloride powder (288 mg/mL) and MAI powder (447 mg/mL) were dissolved in DMF and stirred for 12 h at 60 °C to prepare the PbCl_2_/MAI mixture precursor solution.

The ITO substrates were treated with UV ozone for 15 min before being used. A two-step process was then adopted for the deposition of perovskite films on ITO substrates. All the fabrication processes were conducted in the ambient environment. For MAPbI_3_ perovskite, the PbI_2_ precursor was spin-coated on the ITO substrate at 6000 rpm for 30 s and then annealed at 70 °C for 10 min. The MAI precursor was then spin-coated at 8000 rpm for 10 s and annealed at 100 °C for 10 min. For MAPbI_3–*x*_Br_*x*_, PEDOT:PSS was spin-coated first on the ITO substrate as a hole transport layer at 5000 rpm for 60 s and then annealed at 120 °C for 10 min. The PbI_2_ precursor was then spin-coated at 6000 rpm for 30 s and then annealed at 70 °C for 10 min. The MAI/MABr mixed precursor was spin-coated at 7000 rpm for 10 s and then annealed at 140 °C for 15 min. For MAPbI_3–*x*_Cl_*x*_, PEDOT:PSS was spin-coated first in a similar way to MAPbI_3–*x*_Br_*x*_. A mixed PbCl_2_/MAI precursor was then spin-coated at 7000 rpm for 30 s and then annealed at 100 °C for 35 min. All deposition works were conducted under ambient conditions. Various heat and UV treatment durations were then used to degrade perovskites after the perovskite films were formed. To observe the heat-induced degradation phenomenon, the samples were heated at 85 °C in an ambient environment according to the International Standards IEC 61646 climatic chamber test. The Petri dish was covered with aluminum foil to prevent light soaking during thermal treatment. On the other hand, to observe the UV-induced degradation phenomenon, the samples were directly irradiated without covering them in the ambient environment by 254 nm UV light and the power is 30 W (G30T8 from Light Sources, Inc., USA).

### Characterization

A field emission scanning electron microscope (FEI Nova 200, USA) was used to check the surface morphology of the perovskite films. The crystallinity and stability of the perovskite films were observed by X-ray diffraction with Cu Kα radiation at 25 kV and 40 mA (Bruker D8 Advanced). The data were collected from 10 to 50° (2-theta), with a step size of 0.04°/step and a scan rate of 2°/min. The methyl and amino functional groups were characterized by Fourier transform infrared spectroscopy (PerkinElmer, USA) to investigate the degradation of perovskite films with wavenumbers between 4000 and 650 cm^–1^.

XPS was applied to verify the composition and chemical states of perovskite samples upon exposure to different heat and UV treatment durations. XPS was recorded with a PHI 5000 VersaProbe I system (ULVAC-PHI, Japan) with Al Kα X-ray (25 W, 100 μm). The takeoff angle of the photoelectron was fixed at 45°, and a dual-beam charge neutralizer was used for charge compensation. The XPS spectra were charge referenced to the C 1s peak at ∼284.8 eV. The narrow scan spectra were collected with a pass energy of 23.5 eV. The energy step of the spectra is 0.2 eV. No sputtering was performed prior to analysis.

ToF-SIMS depth profiles were acquired using a PHI TRIFT V nanoTOF (ULVAC-PHI, Japan) system. The acceleration voltage of the analyzed incident C_60_^+^ ion was 20 kV with a beam current of ∼0.08 nA-DC. A pulsed C_60_^+^ (approximately 8200 Hz with a 15 ns pulse length) rastering over a 50 μm × 50 μm area was applied as the primary ion beam for molecular analysis. A 20 kV C_60_^+^ with 1 nA DC beam current rastering over a 500 μm × 500 μm area was used to remove nanoscale perovskite layers.

## Results and Discussion

### Heat-Induced Degradation

**Figure 1:**
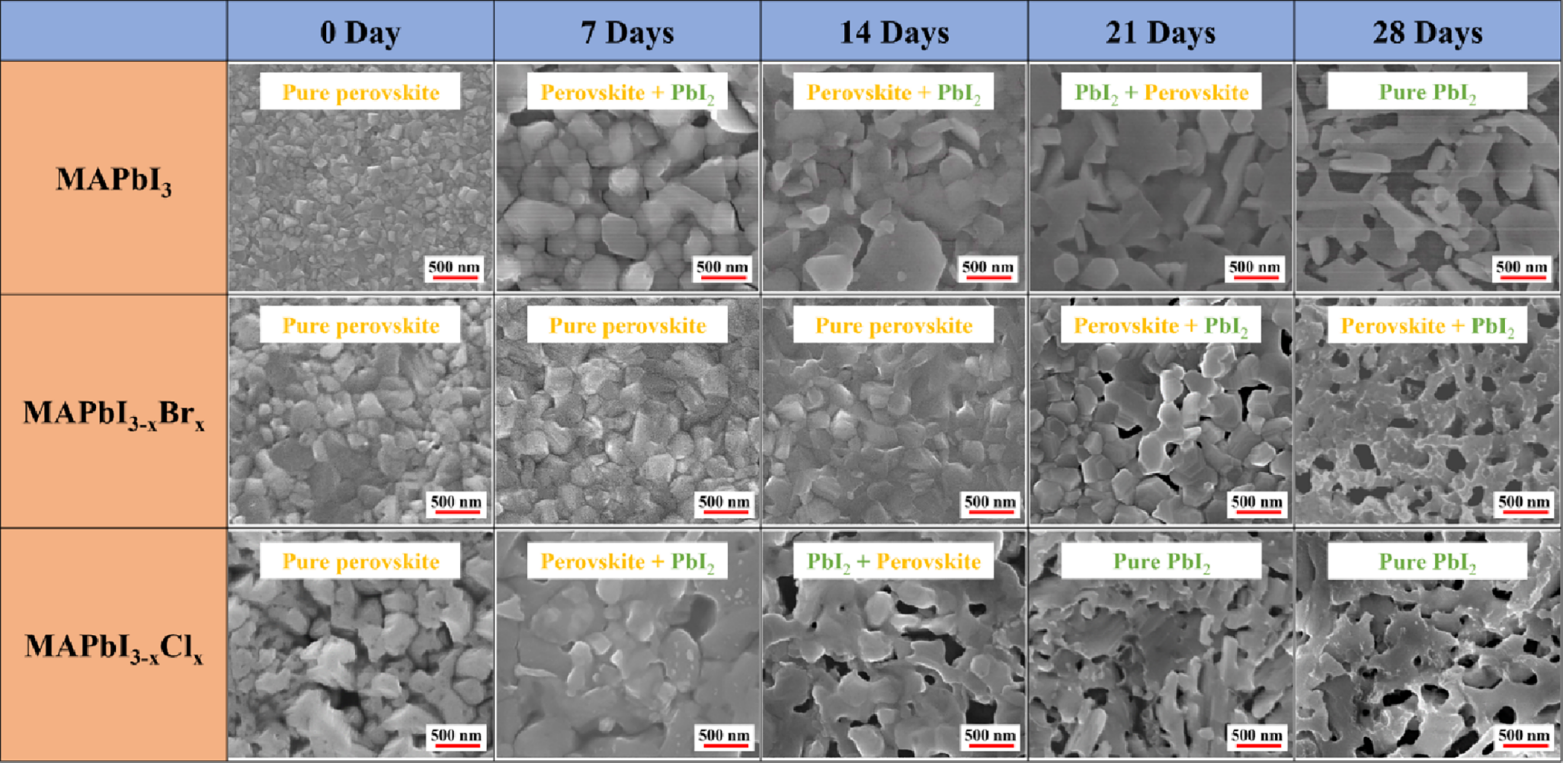
SEM images of MAPbI_3_, MAPbI_3–*x*_Br_*x*_, and MAPbI_3–*x*_Cl_*x*_ perovskite films upon heat exposure.

**Figure 2:**
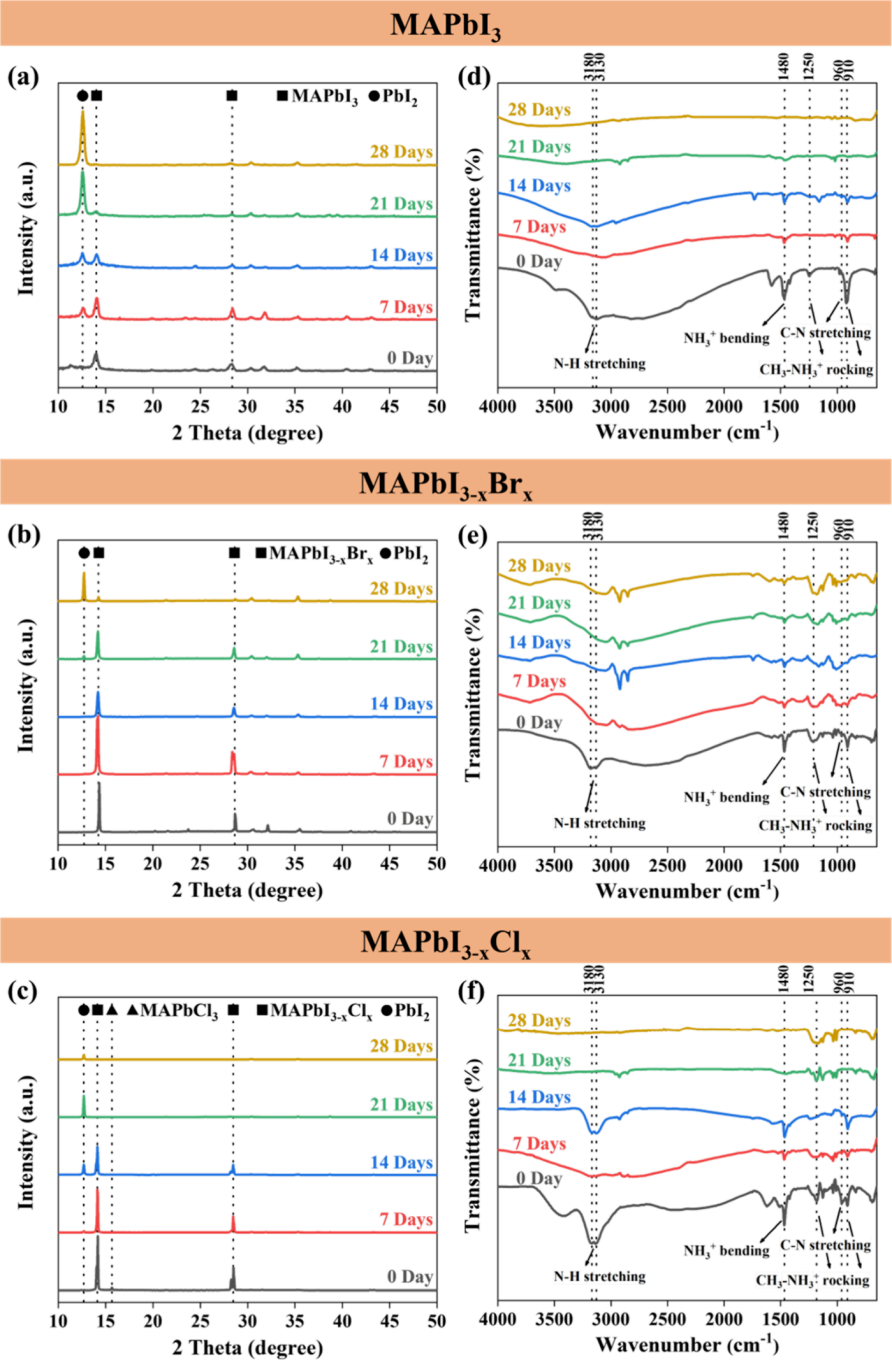
XRD spectra and FTIR spectra of perovskite films upon heat exposure. (a–c) XRD spectra of MAPbI_3_, MAPbI_3–*x*_Br_*x*_, and MAPbI_3–*x*_Cl_*x*_, respectively. (d–f) FTIR spectra of MAPbI_3_, MAPbI_3–*x*_Br_*x*_, and MAPbI_3–*x*_Cl_*x*_, respectively.

SEM was used to examine the surface morphology of perovskite films upon exposure to various thermal durations, as shown in Figure 1. It was found that the 0 day (fresh) MAPbI_3_ film consisted of dense cubic-like crystals. The grain size of MAPbI_3_ became larger after 7 days of thermal treatment due to recrystallization. As the time was prolonged, the crystal boundaries were altered gradually, which indicated the formation of PbI_2_ over time (Figure 2). As shown in the first row in Figure 1, the morphology of the MAPbI_3_ film showed a small number of horizontal flaky crystals at 14 days and changed to vertical sheet-like crystals after 21 days and 28 days of heat treatment. In addition, a large number of holes and gaps between the grains were observed, especially in the 28-day sample. For the MAPbI_3–*x*_Br_*x*_ film, the grain size of the fresh sample was not as uniform as that of the fresh MAPbI_3_ film. A larger recrystallized grain size was observed after a few days of heat treatment, which was similar to the trend in the MAPbI_3_ film. However, the crystals of the MAPbI_3–*x*_Br_*x*_ sample did not change significantly until 21 days of treatment. Coral-like crystals appeared in the MAPbI_3–*x*_Br_*x*_ films at 21 days and 28 days. The MAPbI_3–*x*_Cl_*x*_ film exhibited a very different morphology with many holes/gaps in the fresh sample compared to the others (third row in Figure 1). The grain size of the MAPbI_3–*x*_Cl_*x*_ film also became larger, and coral-like crystals also appeared after long-term heat treatment, which is consistent with the literature.^38−40^

To verify the heat-induced decomposition of perovskite crystals, XRD and FTIR are used to distinguish the crystallinity of perovskites and the functional groups of the organic compounds, as shown in Figure 2. The polycrystalline properties of perovskite films formed by the spin-coating method^61^ are displayed in the XRD spectra in Figure 2a–c. The peak at ∼14° represented the perovskite crystal, and the peak at ∼12.6° represented the crystal of PbI_2_. The fresh MAPbI_3_ sample appeared to consist of a pure perovskite crystal in Figure 2a, with several faint ITO signals.^62^ The PbI_2_ signal appeared in the 7-day sample, and its intensity increased with increasing heat treatment duration. On the other hand, the MAPbI_3_ perovskite signals weakened significantly in 21 days and entirely disappeared in 28 days, which indicated that the MAPbI_3_ completely decomposed to PbI_2_ after 28 days of heat treatment. For the bromine-doped perovskite films, the 0-, 7-, and 14-day samples demonstrated pure perovskite signals with a few ITO signals. The PbI_2_ signal of the MAPbI_3–*x*_Br_*x*_ film appeared in the 21-day sample (Figure 2b). The MAPbI_3–*x*_Br_*x*_ crystal degraded dramatically between 21 days and 28 days since the ratio of MAPbI_3–*x*_Br_*x*_ to PbI_2_ decreased significantly. The fresh chloride-doped perovskite film exhibited a weak signal at ∼15.6°, which represented the MAPbCl_3_ crystal in the (100) plane,^63−65^ as shown in Figure 2c. The PbI_2_ signal of the MAPbI_3–*x*_Cl_*x*_ film appeared at 14 days and turned into the dominant crystal at 21 days, exhibiting the fastest degradation rate among all perovskites. In addition, the PbI_2_ signal decreased at 28 days, possibly due to the escape of I_2_ from the PbI_2_ crystal. From the XRD spectra in Figure 2a–c, the MAPbI_3–*x*_Br_*x*_ crystal demonstrated the best stability against heat followed by the MAPbI_3_ and MAPbI_3–*x*_Cl_*x*_ crystals, which were still observed after 21 days of heat treatment.

The FTIR spectra and characteristic vibrational bands of perovskite films are displayed in Figure 2d–f. The primary peaks refer to different functional groups of organic compounds with various vibrational modes.^66−69^ As demonstrated in Figure 2d–f, the bands are assigned to the CH_3_–NH_3_^+^ rocking at 910 cm^–1^, C–N stretching at 960 cm^–1^, CH_3_–NH_3_^+^ rocking at 1250 cm^–1^, NH_3_^+^ bending at 1480 cm^–1^, and N–H stretching at 3130 and 3180 cm^–1^ for the ammonium functional groups. It is noticed that the spectra of the MAPbI_3_ and the MAPbI_3–*x*_Cl_*x*_ films demonstrated the flat N–H stretching after 7 days of aging compared to the MAPbI_3–*x*_Br_*x*_ film. The possible reasons may be due to high indoor humidity during FTIR measurements. In addition, the MAPbI_3_ and MAPbI_3–*x*_Cl_*x*_ response faster degradation upon exposure to high moisture, which indicates the instability of these two perovskite crystals. All the peaks of MAPbI_3_ entirely disappeared after 28 days (Figure 2d); in contrast, the MAPbI_3–*x*_Br_*x*_ film retained all vibration bands except a weakened peak at 960 cm^–1^ (Figure 2e). For the MAPbI_3–*x*_Cl_*x*_ film, only the peak at 1250 cm^–1^ remained after 28 days of heat treatment, as shown in Figure 2f. In summary, MAPbI_3–*x*_Br_*x*_ demonstrated better stability against heat, which corroborates the information obtained from XRD.

**Figure 3:**
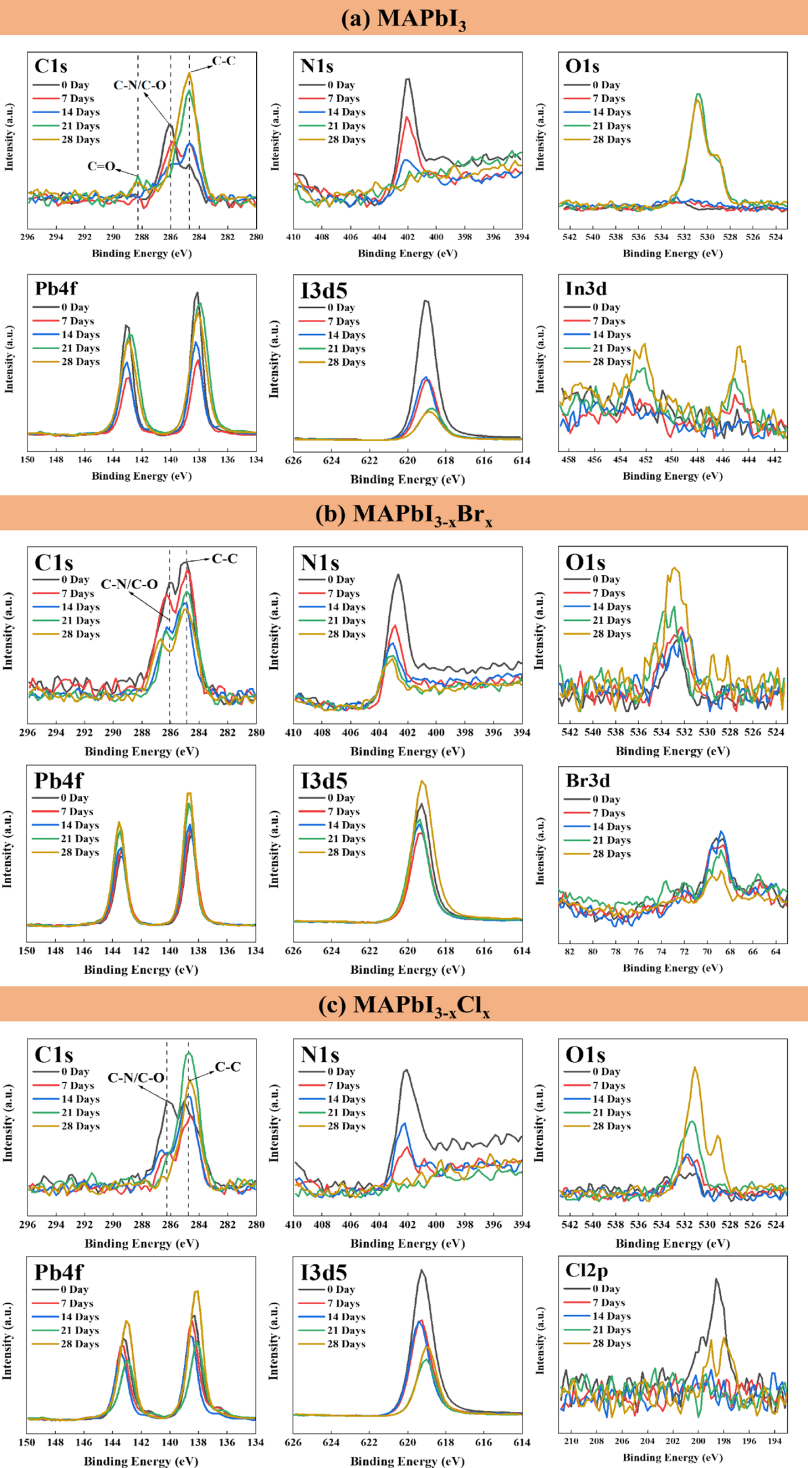
XPS spectra of (a) MAPbI_3_, (b) MAPbI_3–*x*_Br_*x*_, and (c) MAPbI_3–*x*_Cl_*x*_ perovskite films upon heat exposure.

To observe the intrinsic degradation of the perovskite films, the elemental composition and chemical states were further examined by XPS. The XPS spectra of C 1s, N 1s, O 1s, Pb 4f, I 3d5, Br 3d, Cl 2p, S 2p, and In 3d are shown in Figure 3 and Figure S1. It was found that the XPS spectra of all fresh perovskite samples contained a slight O signal, which could be attributed to the fabrication process of the perovskites under ambient conditions. There is no S or In signal from the fresh perovskites, which indicates good coverage of the perovskite films. After heat treatment for various durations, most of the spectra were obviously altered. The curves in the C 1s spectrum of MAPbI_3_ showed that the peak at ∼286 eV (C–N bond) turned to the peak at ∼284.8 eV (C–C bond) and then further to the peak at ∼288.2 eV (C=O bond) over time (Figure 3a). It was observed from the XRD spectra that only PbI_2_ crystals were left after 28 days of heat treatment. A similar phenomenon was confirmed in the N, Pb, and I spectra of the MAPbI_3_ specimen. The N signal disappeared after 21 days, and the peaks of Pb and I shifted slightly to lower binding energies. The peak of Pb also broadened after 21 days of heat treatment. These phenomena indicated that MAPbI_3_ decomposed to PbI_2_, lead oxides, or metallic Pb (Figure 3a). The Pb 4f spectra of MAPbI_3_ show a little hump peak at a lower binding energy (∼136.3 eV), which indicates that metallic Pb was formed during the intermediate stage of the heat-induced decomposition (Figure S1a). The normalized O 1s spectra in Figure S1a also prove the formation of the lead oxide due to the peak shift after a long exposure time. Similar phenomenon is also reported in other literature works.^21,70,71^ The decline in I content could be attributed to the escape of HI_(g)_ and I_2(g)_. It is also well-known that the reversible reaction may occur with a suitable atmosphere for replacing the escape of the gas.^72−74^ In addition, the enhanced In signal indicates the gradual exposure of the ITO substrate. Accordingly, the degradation pathways for MAPbI_3_ can be summarized as follows:
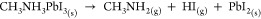
4
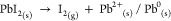
5
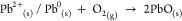
6

**Figure 4:**
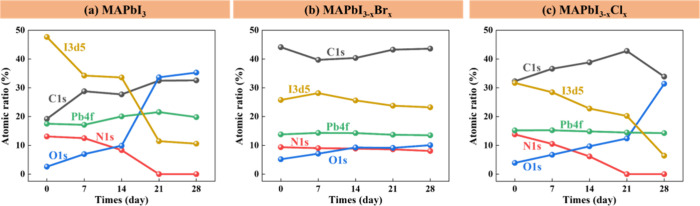
Elemental composition changes of (a) MAPbI_3_, (b) MAPbI_3–*x*_Br_*x*_, and (c) MAPbI_3–*x*_Cl_*x*_ perovskite films during thermal degradation obtained from XPS analysis.

The curves in the C 1s spectrum of MAPbI_3–*x*_Br_*x*_ demonstrated a similar decomposition trend with a higher C–C/C-N ratio over time (Figure 3b). Compared to MAPbI_3_, the MAPbI_3–*x*_Br_*x*_ crystal shows a slower degradation rate due to additional bromine doping. This phenomenon could also be proven by the decreasing intensity of the N peak of MAPbI_3–*x*_Br_*x*_. The quantity of Br in the MAPbI_3–*x*_Br_*x*_ crystal decreased over time but still existed in sufficient amounts to retain the perovskite crystal in 28 days (XRD in Figure 2b). The broadening peak of Pb and I could be observed after 28 days of heat treatment, indicating that complex compounds might form due to the degradation of perovskite crystals. Comparing the curves of Br and I, the content of I decreased faster than that of Br after 7 days of heat treatment. Although the signals of Br and I seem to increase after long-term thermal degradation in Figure 3b, the quantification in Figure 4b and Figure S2b shows a decline in the content of Br and I. This implied that the remaining perovskite is a Br-rich crystal. Furthermore, a shoulder peak at ∼529 eV in the O 1s spectra indicates the formation of amorphous PbO after long-term exposure to heat. In addition, few S and In signals appeared at 28 days, indicating that the perovskites did not totally degrade and still covered the substrate well (Figure S1b). The degradation pathways for MAPbI_3–*x*_Br_*x*_ are summarized below. It should be noted that the value for *y* is higher than *x* in eq 7. Equations 8 and 9 are identical to eqs 5 and 6, respectively.
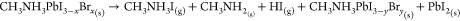
7
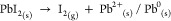
8
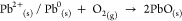
9

The MAPbI_3–*x*_Cl_*x*_ crystal also shows the C–N bond to the C–C bond with a faster transition compared to MAPbI_3_ (Figure 3c). In addition, the N and Cl signals disappeared at 21 days, and the peaks of Pb and I shifted slightly to the right, which indicated the decomposition of the MAPbI_3–*x*_Cl_*x*_ crystal. It is worth noting that the I content was larger than the Cl content after 7 days of heat treatment, which indicated that MAPbI_3_ might appear as the intermediate product during the degradation process before the total decomposition of the MAPbI_3–*x*_Cl_*x*_ crystal. The enhanced S signal also indicated the gradual exposure of the PEDOT:PSS layer (Figure S1c). All these results confirmed that the chlorine-doped perovskites demonstrated the poorest stability to heat. The degradation pathways for MAPbI_3–*x*_Cl_*x*_ can be summarized as follows:
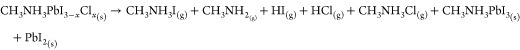
10
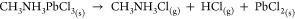
11
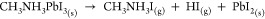
12
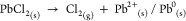
13
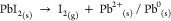
14
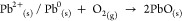
15

The degradation process described by eq 11 is due to the observation of phase separation in MAPbI_3–*x*_Cl_*x*_ perovskite (Figure 2c). Equations 14 and 15 are identical to eq 5 and 6, respectively.

The composition changes of MAPbI_3_, MAPbI_3–*x*_Br_*x*_, and MAPbI_3–*x*_Cl_*x*_ perovskite films are depicted in Figure 4 and Figure S2. The atomic ratios of C, Pb, and O in MAPbI_3_ rise with prolonged exposure to heat. Specifically, carbon gradually increases from 19.19 to 32.58%. Oxygen experiences a significant rise from 2.58 to 35.28% after 28 days of heating. The atomic ratio of Pb also generally increases; after 21 days of heating, Pb increases from 17.49% to its peak at 21.55% and then slightly decreases to 19.8% by the 28 days of heating. The compositions of C, Pb, and O in MAPbI_3_ increase, whereas the atomic ratios of N and I decrease. The significant decline in N and I implied the escape of MAI_(g)_, HI_(g)_, and I_2(g)_ as degradation products from the MAPbI_3_ crystal. The enhanced In signal also indicated the gradual exposure of the ITO substrate. For MAPbI_3–*x*_Br_*x*_, the content of I, N, and Br scarcely decrease. In addition, barely any S and In signals appeared, which indicated that MAPbI_3–*x*_Br_*x*_ hardly degraded and still covered the substrate well. On the other hand, the ratios of I, N, and Cl in the MAPbI_3–*x*_Cl_*x*_ sample decreased significantly. The exposure of the PEDOT:PSS layer underneath was proven by the enhancement of the S signal, implying the decomposition of the MAPbI_3–*x*_Cl_*x*_ crystal. In summary, MAPbI_3–*x*_Br_*x*_ demonstrated better stability, with consistent data shown in SEM, XRD, and FTIR.

### UV-Induced Degradation

**Figure 5:**
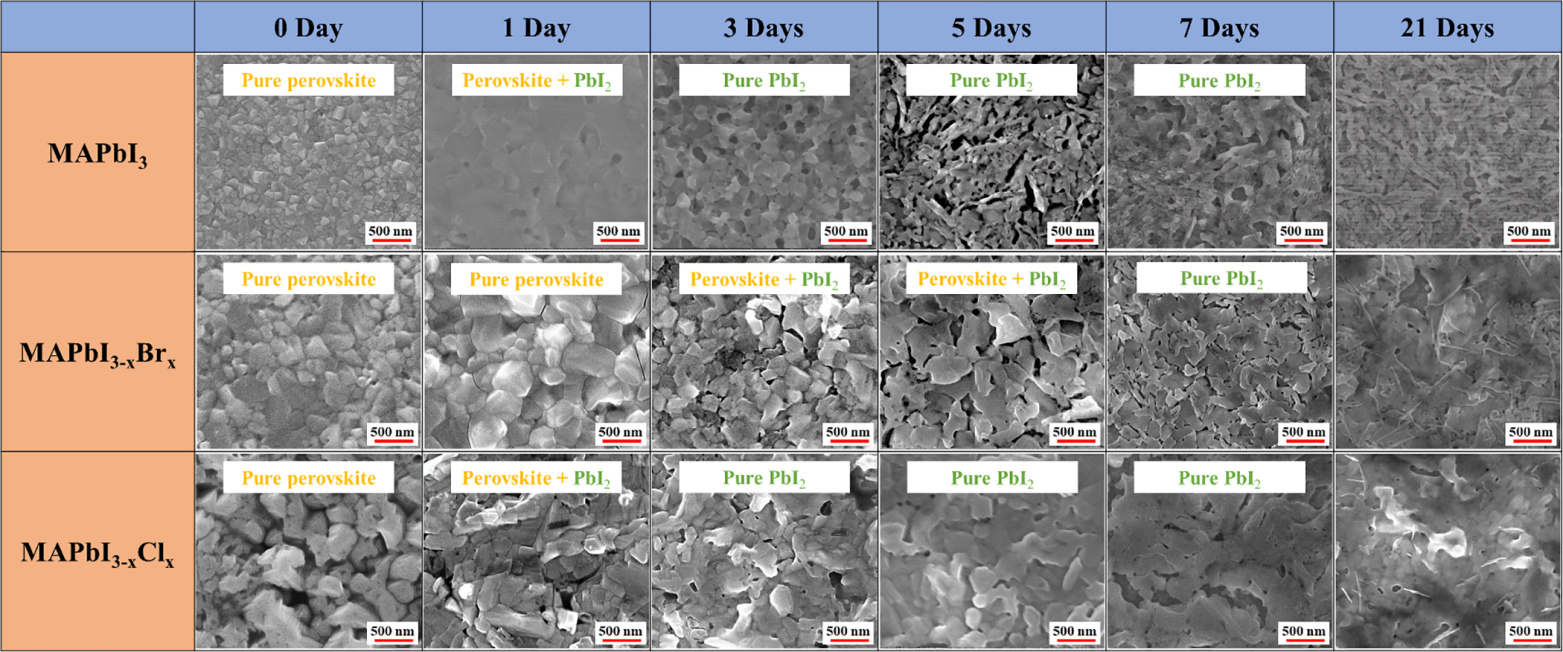
SEM images of MAPbI_3_, MAPbI_3–*x*_Br_*x*_, and MAPbI_3–*x*_Cl_*x*_ perovskite films upon UV light exposure.

**Figure 6:**
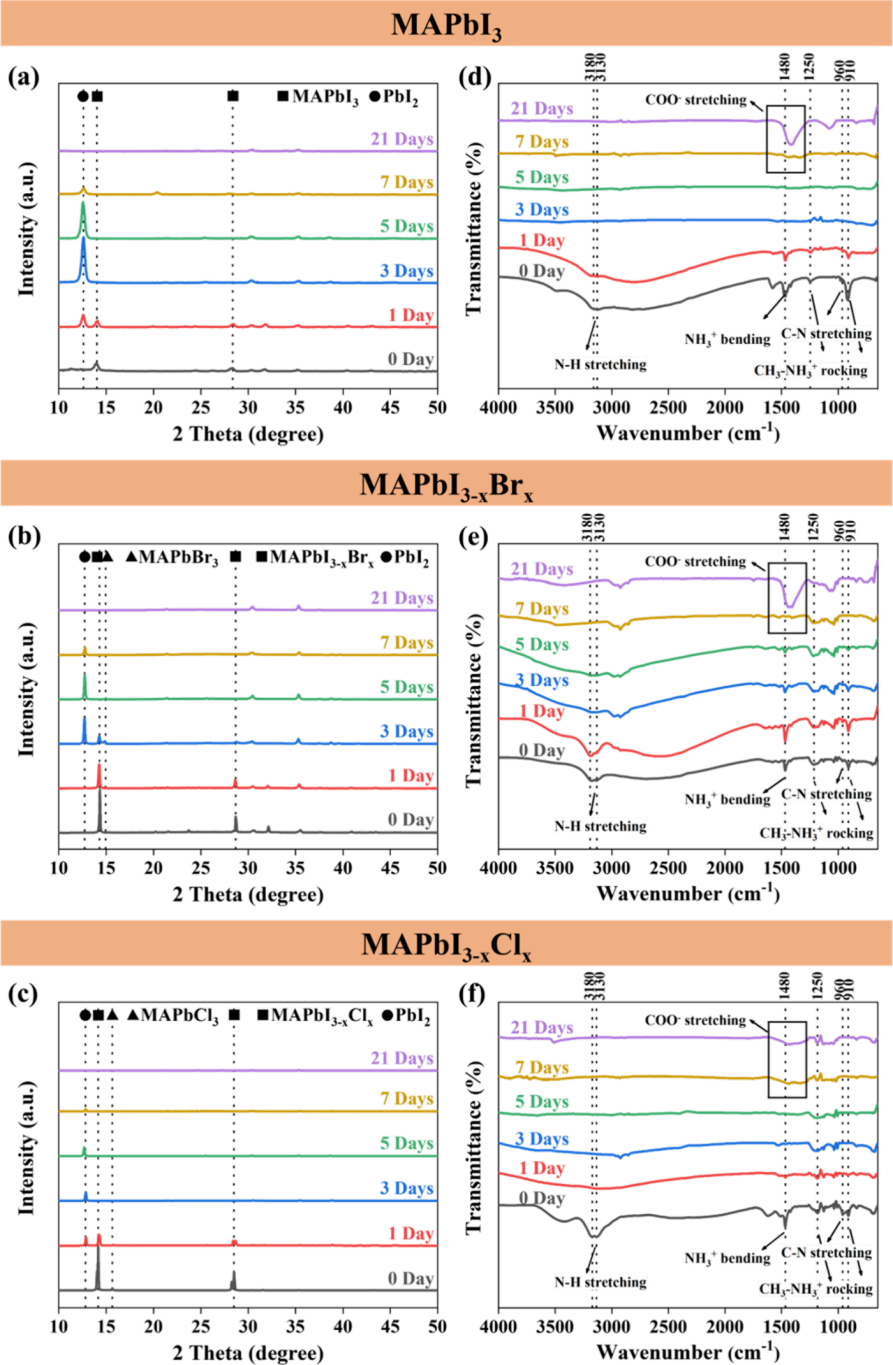
XRD spectra and FTIR spectra of perovskite films upon UV light exposure. (a–c) XRD spectra of MAPbI_3_, MAPbI_3–*x*_Br_*x*_, and MAPbI_3–*x*_Cl_*x*_, respectively. (d–f) FTIR spectra of MAPbI_3_, MAPbI_3–*x*_Br_*x*_, and MAPbI_3–*x*_Cl_*x*_, respectively.

The surface morphology was examined by SEM to observe the change in perovskite films upon UV light exposure for different durations, as shown in Figure 5. The grain size of MAPbI_3_ became larger and flat after UV degradation for 1 day. Some holes between the grains gradually appeared, and the cubic crystals turned into sheet-like and needle-like crystals with longer aging times. The surface morphology dramatically changes from the round-shaped to the needle-like crystal between 3 and 5 days of UV exposure. The crystallinity result obtained by XRD further confirmed that both samples are pure PbI_2_ crystals (Figure 6a). At 21 days, the original MAPbI_3_ structure collapsed, and most of the grains presented needle-like crystals, which implied total decomposition of the MAPbI_3_ crystal. The corresponding crystal structure was confirmed by XRD, as shown in the next section. The overall decomposition processes of these three kinds of perovskite films were similar: recrystallized grain growth occurred followed by structural collapse. Most perovskite grains presented a large number of holes and gaps with needle-like crystals after 21 days of UV exposure. Interestingly, it was found that the surface morphology change rate of the MAPbI_3–*x*_Br_*x*_ crystal was the slowest, while the MAPbI_3–*x*_Cl_*x*_ crystal presented the most obvious morphology change. In comparison to heat-induced degradation, UV light destroys perovskite crystals faster.

The XRD and FTIR spectra provide information on crystallinity and organic functional groups on the UV-induced degradation of perovskite films, as shown in Figure 6. All the fresh samples demonstrated strong perovskite signals at ∼14° in the XRD spectra (Figure 6a–c). PbI_2_ signals appeared after 1 day of UV exposure for the MAPbI_3_ and MAPbI_3–*x*_Cl_*x*_ crystals. The PbI_2_ signal appeared in the MAPbI_3–*x*_Br_*x*_ sample until 3 days of UV exposure. The results indicated that the MAPbI_3–*x*_Br_*x*_ crystal has the best UV stability among these perovskites. In addition, a peak at ∼15° related to MAPbBr_3_^75^ appeared simultaneously in the MAPbI_3–*x*_Br_*x*_ sample under 3 days of UV exposure, which indicated the appearance of separate phases (MAPbI_3–*x*_Br_*x*_ and MAPbBr_3_) during the decomposition process. The PbI_2_ signals eventually disappeared and the structure became completely amorphous after long-term UV exposure for all perovskites.

The FTIR spectra illustrated the characteristic vibrational bands of the perovskite films in Figure 6d–f. The absorption peaks at 3180, 3130, 1480, 1250, 960, and 910 cm^–1^ represented the N–H stretching, NH_3_^+^ bending, CH_3_–NH_3_^+^ rocking, C–N stretching, and CH_3_–NH_3_^+^ rocking, respectively. As the aging time increased, all the peaks in the MAPbI_3_ sample disappeared almost completely in 3 days. A similar situation occurred for MAPbI_3–*x*_Cl_*x*_ samples, where the peaks at 1480, 960, and 910 cm^–1^ disappeared, and the peak at 1250 cm^–1^ remained for 3 days. Despite being weaker, all the peaks of the MAPbI_3–*x*_Br_*x*_ crystal remained after 5 days of UV treatment, which also implied that MAPbI_3–*x*_Br_*x*_ had better UV stability than the others. In summary, the FTIR spectra in Figure 6d–f could be considered to verify the crystallinity information in the XRD spectra since they indicated consistent conclusions. The new formation on COO^–^ stretching vibrations indicated that new amorphous compounds were formed (verified by XRD spectra) after 7 (MAPbI_3–*x*_Cl_*x*_) and 21 (MAPbI_3_ and MAPbI_3–*x*_Br_*x*_) days of UV exposure.

**Figure 7:**
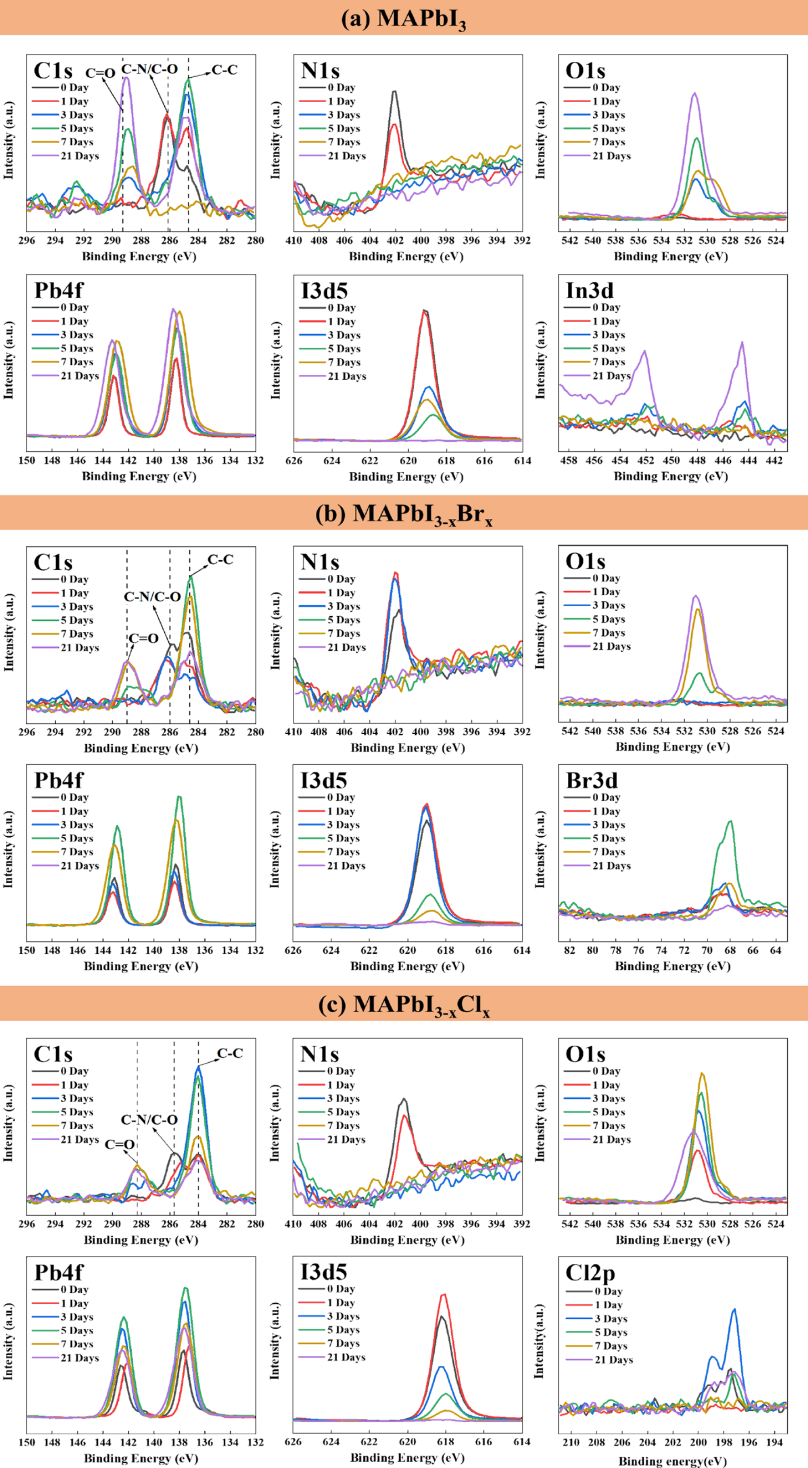
XPS spectra of (a) MAPbI_3_, (b) MAPbI_3–*x*_Br_*x*_, and (c) MAPbI_3–*x*_Cl_*x*_ perovskite films upon UV light exposure.

To observe the intrinsic UV-induced degradation of the perovskites, the chemical state and composition of the samples were also examined by XPS. Figure 7 and Figure S3 illustrate the XPS spectra of C 1s, N 1s, O 1s, Pb 4f, I 3d5, Br 3d, Cl 2p, S 2p, and In 3d from perovskites with various UV light exposure durations. The fresh C 1s spectrum (0 day) displayed a main peak at 286.1 eV, which represented the C–N bond of MAPbI_3_. A shoulder peak was observed at ∼285 eV and represented the C–C bond of perovskite and surface contamination. It was clear that the intensity of the C–N peak gradually decreased and more C–C and C=O (∼289 eV) bonds formed with increasing UV exposure time. The significant formation of the C=O bond reflected the severe decomposition of the perovskite crystal damaged by UV light. The disappearance of the N 1s peak at ∼402 eV proved that the organic compounds escaped over time (3 days of exposure), which was consistent with the XRD and FTIR spectra in Figure 6. Interestingly, it is also noticed that the O 1s spectra in MAPbI_3_ showed a shoulder peak at ∼529 eV, which indicated the formation of metallic oxide after 3 days of UV exposure. The strong O 1s spectra also indicate the formation of C=O observed in the C 1s spectrum. The generation of lead oxide and C=O results in a dramatic change in the surface morphology of PbI_2_ crystals, as observed in Figure 5. The broadened Pb 4f spectra confirmed the formation of the lead oxide observed in the O 1s spectra. The fading intensity of the I 3d5 spectra confirmed the structural collapse of the MAPbI_3_ crystal, accompanied by an increasing indium signal due to substrate exposure. Therefore, the degradation pathways for MAPbI_3_ under UV-light illumination follow eqs 4 to 6, similar to the degradation pathways for MAPbI_3_ under heat treatment.

The XPS spectra of the MAPbI_3–*x*_Br_*x*_ perovskite demonstrated a slower chemical change than that of MAPbI_3_. For example, a strong N 1s peak was still observed in the sample with 3 days of UV exposure, which indicated the presence of the perovskite crystal. In addition, the slowest appearance of the O 1s peak in MAPbI_3–*x*_Br_*x*_ perovskite confirmed the robustness of the crystal structure compared to the other two perovskites. Significant losses of N and I were observed until 5 days of UV exposure, reflecting the escape of organic compounds from the crystal. The presence of Br helps to stabilize the perovskite crystal structure due to the shorter atomic distance,^76^ resulting in the highest intensity in the 5-day Br 3d spectrum (Figure S4). A possible explanation is that the bromine compounds were distributed in the deeper region of the perovskite structure and moved to the surface as decomposition occurred. The severe decline in the I signal might also have increased the content of Br. The degradation pathways can be summarized as follows:
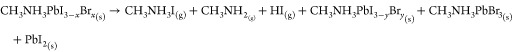
16
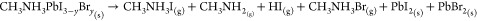
17
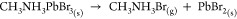
18
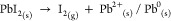
19
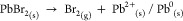
20
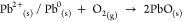
21

The value for *y* is higher than that for *x* in eq 16. Equations 19 and 21 are identical to eqs 5 and 6, respectively.

**Figure 8:**
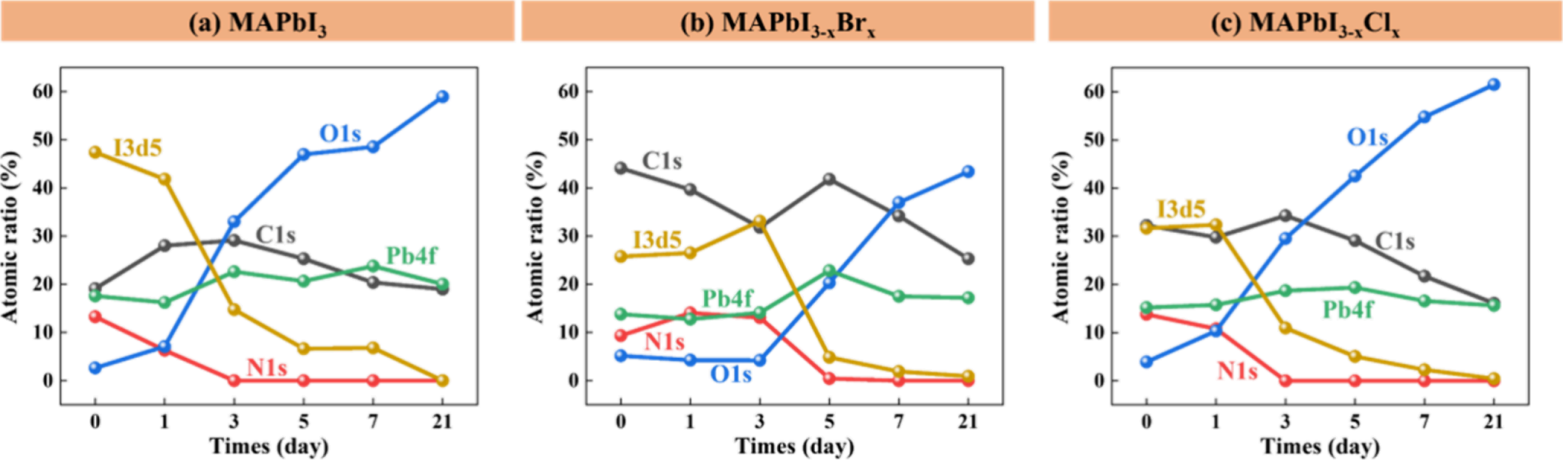
Elemental composition changes of (a) MAPbI_3_, (b) MAPbI_3–*x*_Br_*x*_, and (c) MAPbI_3–*x*_Cl_*x*_ perovskite films during UV-light-induced degradation obtained from XPS analysis.

The XPS spectra of the MAPbI_3–*x*_Cl_*x*_ crystal were similar to the MAPbI_3_ spectra. The Cl 2p signal fluctuated during degradation (Figure S4), which might be attributed to a significant increase in oxygen and the great loss of I 3d5 and C 1s under ambient conditions (Figure 7 and Figure 8). The degradation pathways for MAPbI_3–*x*_Cl_*x*_ under UV-light illumination follow eq 10 to 15, with similar degradation pathways for MAPbI_3–*x*_Cl_*x*_ under heat treatment, although some of the above reactions are reversible by providing a suitable atmosphere (reactions 16–18). However, as PbI_2_ degrades into Pb^0^ and I_2_ and further forms the PbO compound upon exposure to UV light, the reversible reaction should no longer be considered (reactions 19−21). The severe crystal structure collapse results in permanent damage of materials.^72^

The composition evolution of MAPbI_3_, MAPbI_3–*x*_Br_*x*_, and MAPbI_3–*x*_Cl_*x*_ perovskite films over time is depicted in Figure 8 and Figure S4. The amounts of N and I in the MAPbI_3_ sample decreased significantly over time, indicating the escape of the organic compounds MAI, HI, and I_2_. The increase in the In signal also implied the exposure of the ITO substrate (Figure S4a). On the other hand, the decreasing rates of N, I (Figure 8b), and Br (Figure S4b) in the MAPbI_3–*x*_Br_*x*_ crystal were slower. The quantity of I and N in 3 days and that of Br in 5 days remained sufficient to indicate the retention of perovskite structures (Figure 6b). The S signal appeared after 5 days of UV treatment, which indicated the exposure of PEDOT:PSS underneath the perovskite film. The MAPbI_3–*x*_Cl_*x*_ crystal presented decomposition progress similar to that of the MAPbI_3_ crystal. The ratios of N and I in the MAPbI_3–*x*_Cl_*x*_ sample also decreased significantly. The Cl content fluctuated during degradation. The enhanced S signal under 3 days of UV treatment indicated the exposure of the PEDOT:PSS layer as well. In summary, MAPbI_3–*x*_Br_*x*_ demonstrated better stability under exposure to UV light. Generally, the incorporation of Br ions assists in elevating the Goldschmidt tolerance factor, reaching approximately 0.9. This particular value of the tolerance factor can lead to the stabilization of the α-phase cubic structure.^77,78^ Cl ions are also known to enhance the tolerance factor, although an additional MAPbCl_3_ phase is observed in XRD analysis. This occurrence could be ascribed to the ambient environment during the synthesis process of the material. Consequently, Cl has a lesser impact on maintaining the MAPbI_3–*x*_Cl_*x*_ phase. The data obtained from SEM, XRD, FTIR, and XPS are consistent. Furthermore, all three perovskite crystals demonstrated worse stability under UV-light illumination than heat treatment. The strong O 1s spectra of the perovskite also indicated that UV light facilitates the oxidation process and damages crystals more severely than heat.

**Figure 9:**
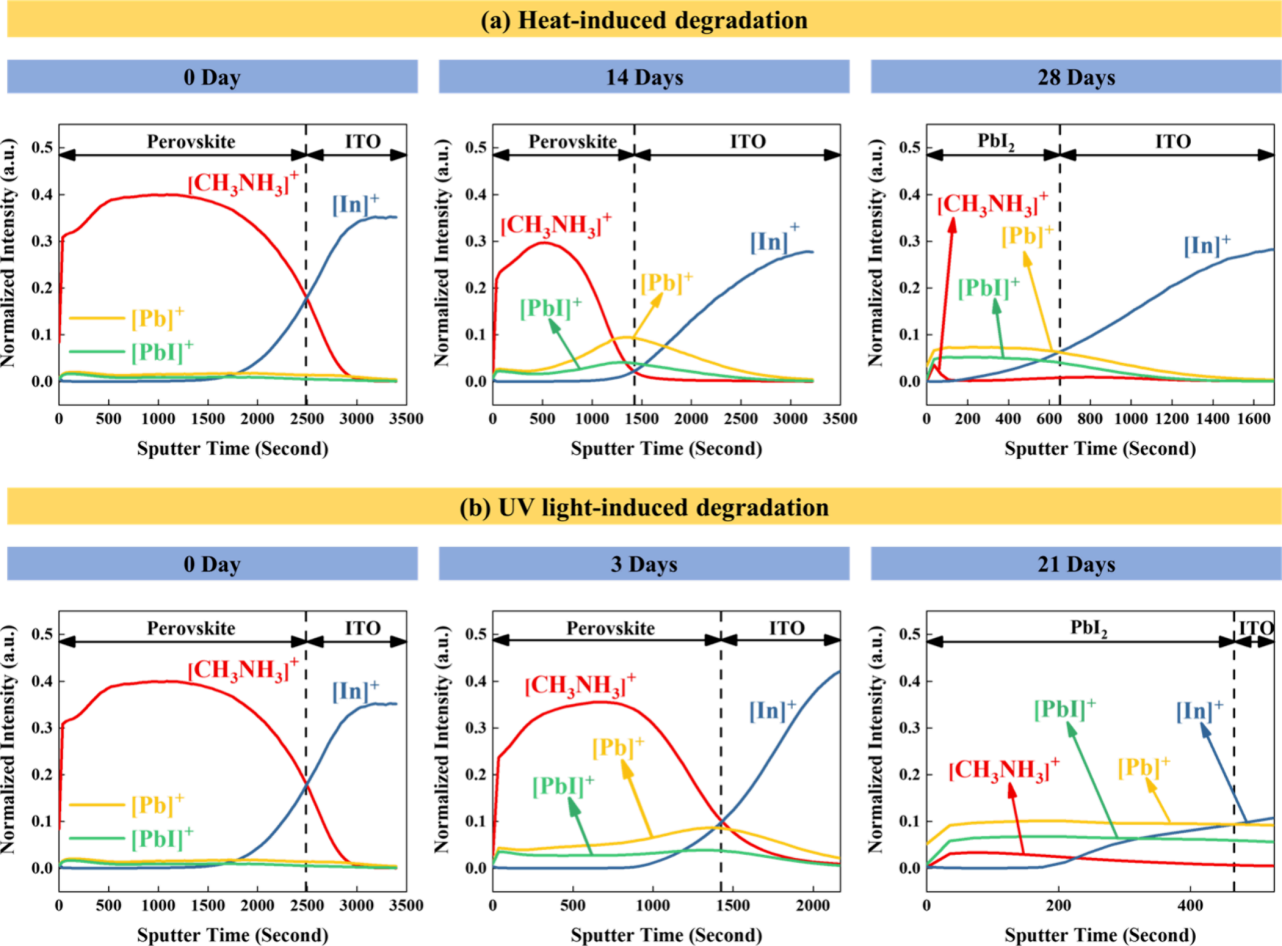
Depth profiles of MAPbI_3–*x*_Br_*x*_ perovskite films after (a) heat exposure and (b) UV-light exposure, acquired by ToF-SIMS.

The MAPbI_3–*x*_Br_*x*_ crystal demonstrated the best stability against heat and UV light among the three kinds of perovskite crystals; hence, ToF-SIMS was introduced to further reconstruct the molecular depth profiles of the MAPbI_3–*x*_Br_*x*_ crystal during the decomposition process. According to the results from the XRD spectra, 0-, 14-, and 28-day heat treatment samples were chosen to study the molecular distribution after aging. The fresh specimen and the 3- and 21-day UV aging samples were chosen for comparison. The distribution of the positive ions [CH_3_NH_3_]^+^, [Pb]^+^, [PbI]^+^, [PbO]^+^, [PbSO]^+^, [PbBr]^+^, and [In]^+^ represent perovskite/PEDOT:PSS/ITO interfacial regions, as shown in Figure 9 and Figure S5. The measurement of [PbSO]^+^ ions was used to represent the PEDOT:PSS layer. The interface of the perovskite layer and the ITO layer was determined by the intersection region of [CH_3_NH_3_]^+^ and [In]^+^, as shown in Figure 9a. Thus, the fresh MAPbI_3–*x*_Br_*x*_ thin film showed a two-layer structure. This 0-day ToF-SIMS result confirms the formation of MAPbI_3–*x*_Br_*x*_ crystals with a good mixture of PbI_2_ and MAI/MABr precursors under a two-step process. Other signals, such as [Pb]^+^, [PbI]^+^, [PbBr]^+^, and [PbO]^+^, were weak in the fresh sample (0 day). The thickness of the perovskite layer decreased as the heat treatment time increased. Moreover, the [Pb]^+^, [PbI]^+^, and a small amount of [PbBr]^+^ aggregated at the interface between perovskite and the ITO substrate after 14 days of thermal treatment. Compared to the fresh sample, in the initial degradation stage, [Pb]^+^ and [PbI]^+^ were more concentrated at the outermost surface of the perovskite crystal. Therefore, CH_3_NH_3_I_(g)_ and HI_(g)_ leached from the surface of the MAPbI_3–*x*_Br_*x*_ crystal (reaction 7), and [Pb]^+^, [PbI]^+^, and [PbBr]^+^ (byproduct of the heat-induced degradation) migrated toward the ITO side, triggered by the heat source underneath the substrate. The movement of MAI toward the surface of the specimen also participated in refilling vacancies left by CH_3_NH_3_I_(g)_ and HI_(g)_ leaching, resulting in the accumulation of [Pb]^+^, [PbI]^+^, and [PbBr]^+^. Therefore, the heat-induced decomposition of the MAPbI_3–*x*_Br_*x*_ crystal occurred by molecular migration in the opposite direction. After 28 days of heat treatment, only [Pb]^+^ and [PbI]^+^ were left on the surface, and [PbBr]^+^ infiltrated into the ITO layer.

Interestingly, [Pb]^+^ and [PbI]^+^ were distributed more uniformly after 3 days of UV exposure. The accumulation of [Pb]^+^, [PbI]^+^, and [PbBr]^+^ at the interface region was still observed. [Pb]^+^ and [PbI]^+^ were the major residues on the surface after 21 days of UV treatment as well. Therefore, it could be speculated that the degradation process begins at the interface between the perovskite layer and the ITO layer. Although the [PbO]^+^, [PbSO]^+^, and [PbBr]^+^ signals in Figure S5 are much weaker than the [CH_3_NH_3_]^+^, [In]^+^, [Pb]^+^, and [PbI]^+^ signals, [PbBr]^+^ shows a much stronger intensity at the interface, which is direct evidence of the decomposition of the MAPbI_3–*x*_Br_*x*_ crystal. Comparing the [PbBr]^+^ signal to [Pb]^+^ and [PbI]^+^ signals showed that [PbBr]^+^ also aggregated at the interface and became distributed in deeper regions. After long-term exposure to both heat and UV light, a thin [PbO]^+^ layer was observed at the outermost surface (Figure S5).

## Conclusions

In conclusion, we have summarized the investigations of the heat- and UV-induced degradation processes of three mixed-halide perovskite crystals (MAPbI_3_, MAPbI_3–*x*_Br_*x*_, and MAPbI_3–*x*_Cl_*x*_) under ambient conditions. The results demonstrate that the three perovskite films are not stable under exposure to heat and UV light. The loss of methylamine (CH_3_NH_2_) is an important indicator in determining the decomposition process of the perovskite crystal. Therefore, the evolution of nitrogen concentration shown by XPS confirms that MAPbI_3–*x*_Br_*x*_ perovskite exhibits the best stability upon exposure to heat and UV light. The addition of Br ions facilitates an increase in the Goldschmidt tolerance factor, reaching approximately ∼0.9. This particular value of the tolerance factor can lead to the attainment of a sturdy α-phase cubic structure. The XPS spectra also reveal that the chemical state evolution differs between heat- and UV-induced degradation. The XRD and XPS results verify that UV light causes faster damage to perovskite crystals than heat treatment. ToF-SIMS proves the degradation mechanism from the perspective of the molecular distribution in the MAPbI_3–*x*_Br_*x*_ specimen. The evaporation of methylamine and HI toward the specimen surface significantly reduces the thickness of the MAPbI_3–*x*_Br_*x*_ thin film. Solid-state [Pb]^+^, [PbI]^+^, and [PbBr]^+^ migrate toward the substrate, which directly shows the decomposition process of the MAPbI_3–*x*_Br_*x*_ perovskite upon exposure to heat and UV light.

## Data Availability

All data generated or analyzed during this study are included in this published article and its Supporting Information. The raw data is available upon request.

## Supporting Information Available

Additional XPS spectra of perovskites upon heat exposure; evolution of heat-degraded perovskite samples in terms of In, Br, S, and Cl; XPS spectra of perovskites upon UV light exposure; evolution of UV-degraded perovskite samples in terms of In, Br, S, and Cl; molecular distribution obtained by ToF-SIMS (PDF)
