# Supplementary material for: Unraveling the Heat- and UV-Induced Degradation of Mixed Halide Perovskite Thin Films via Surface Analysis Techniques
Source: Langmuir. 2024 May 23;40(23):11873–87. doi: 10.1021/acs.langmuir.3c03816 (PMC11171447; doi:10.1021/acs.langmuir.3c03816)
Supplement: Supplementary file 1 — la3c03816_si_001.pdf [file la3c03816_si_001.pdf]

Supporting Information

Unraveling the heat and UV induced degradation of the mixed halide perovskite thin films via surface analysis techniques

*Pei-Chen Huang, Ting-Jia Yang, Chia-Jou Lin, Man-Ying Wang, Wei-Chun Lin**

Department of Photonics, National Sun Yat-sen University, Kaohsiung City 80424, Taiwan (R.O.C.)

E-mail: wclin@mail.nsysu.edu.tw

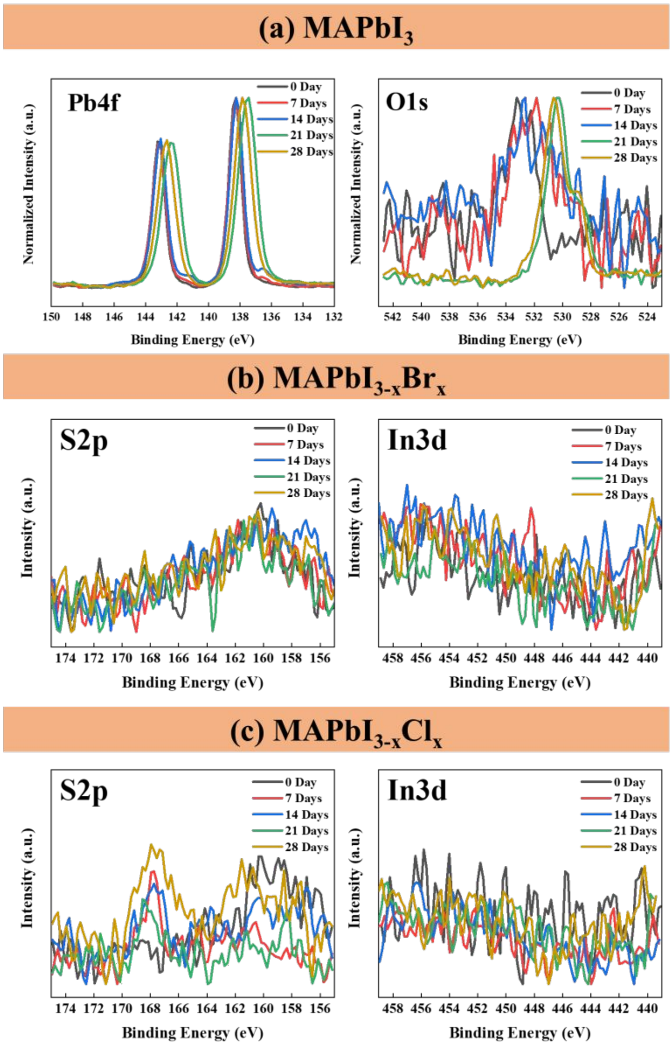

Supplementary Figure 1. XPS spectra of $MAPbI_3$, $MAPbI_{3-x}Br_x$ and $MAPbI_{3-x}Cl_x$ perovskite films upon heat exposure.

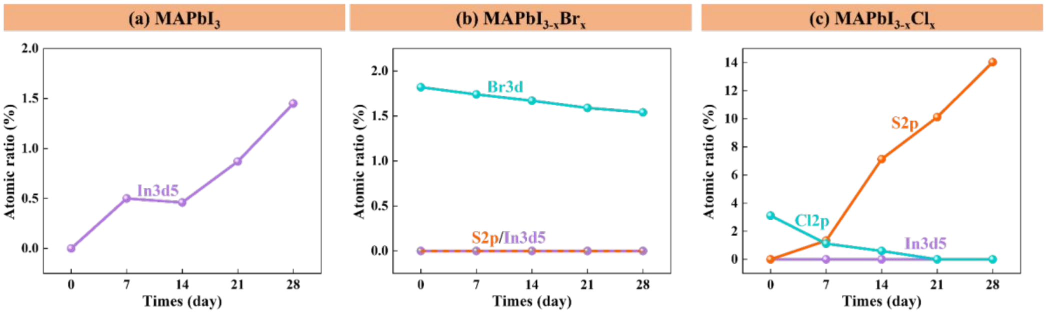

Supplementary Figure 2. The elemental composition change of $MAPbI_3$, $MAPbI_{3-x}Br_x$ and $MAPbI_{3-x}Cl_x$ perovskite films upon heat exposure obtained from XPS analysis.

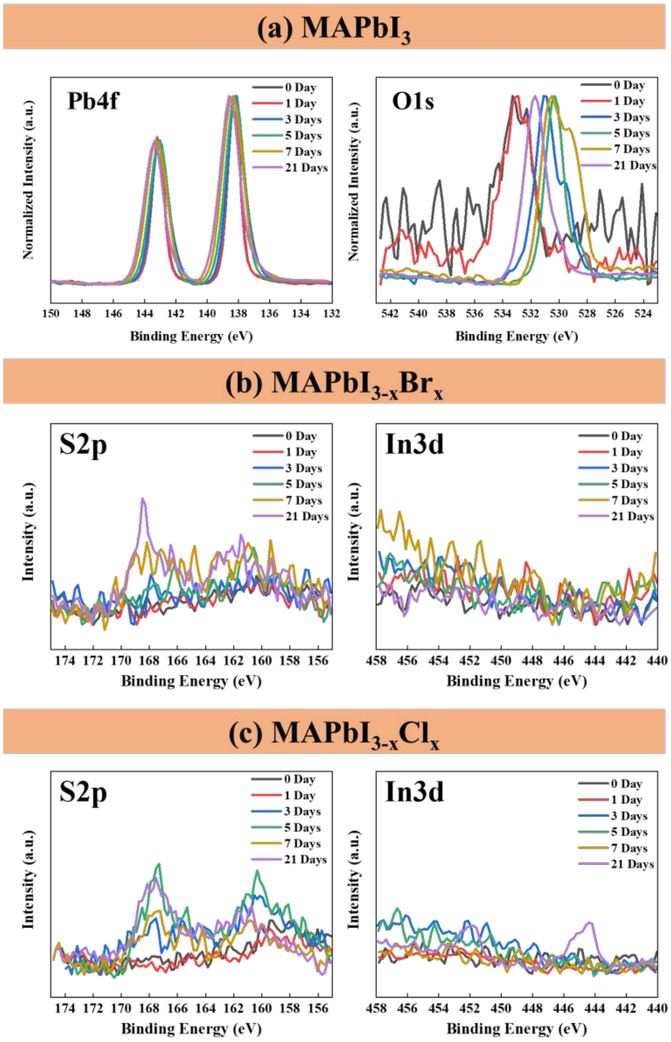

Supplementary Figure 3. XPS spectra of $MAPbI_3$, $MAPbI_{3-x}Br_x$ and $MAPbI_{3-x}Cl_x$ perovskite films upon UV light exposure.

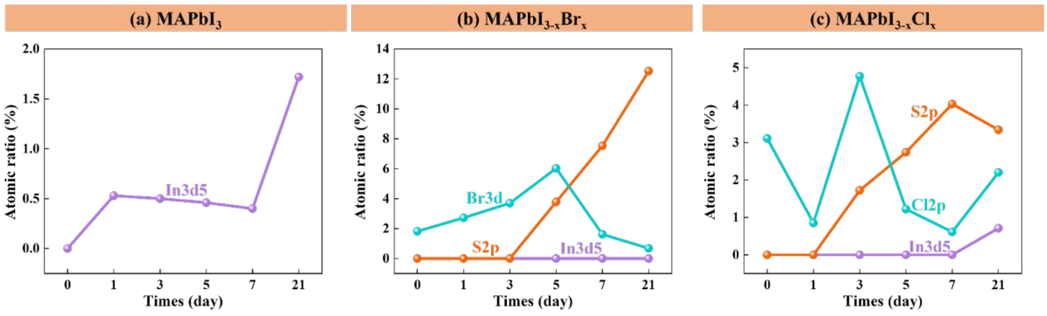

Supplementary Figure 4. The elemental composition change of $MAPbI_3$, $MAPbI_{3-x}Br_x$ and $MAPbI_{3-x}Cl_x$ perovskite films upon UV light exposure obtained from XPS analysis.

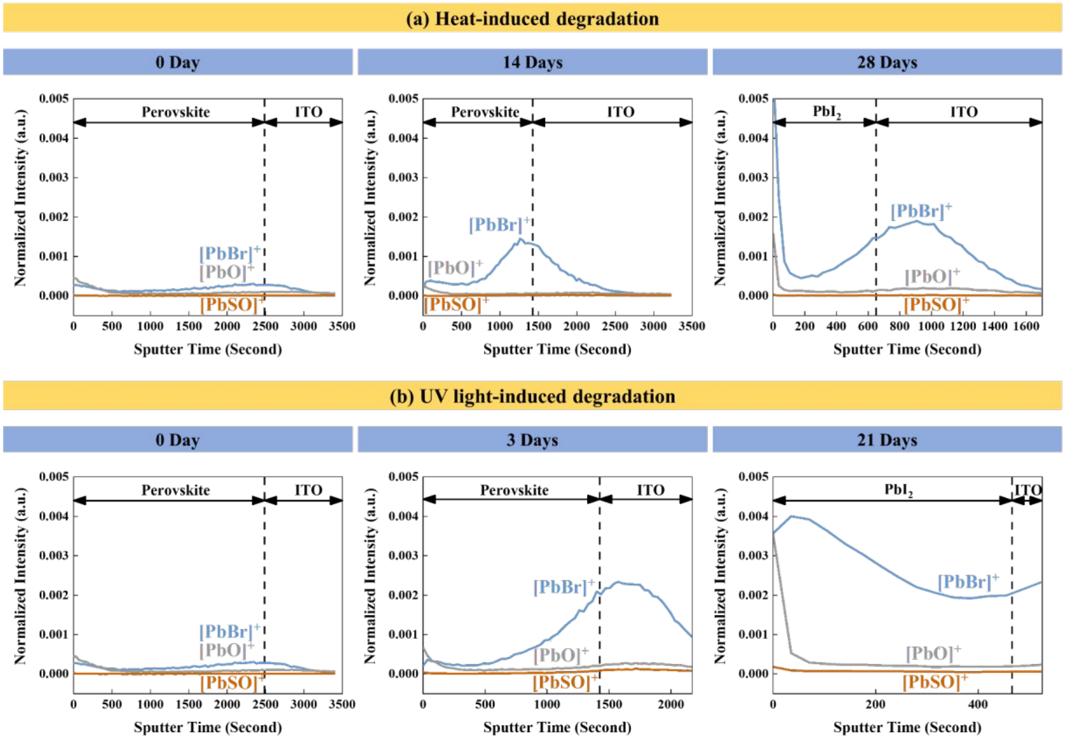

Supplementary Figure 5. The depth profiles of $MAPbI_{3-x}Br_x$ perovskite films upon heat exposure and UV-light exposure acquired by ToF-SIMS, respectively.
